# Research on refractive power calculation formula of intraocular lens based on the principle of double thick lens imaging

**DOI:** 10.3389/fmed.2026.1848131

**Published:** 2026-05-14

**Authors:** Mengdi Yan, Juntao Zhang, Shiming Wang

**Affiliations:** 1 Department of Ophthalmology, Ningbo Municipal Hospital of Traditional Chinese Medicine, Affiliated Hospital of Zhejiang Chinese Medical University, Ningbo, Zhejiang, China; 2 Ophthalmology Center, The Affiliated People’s Hospital of Ningbo University, Ningbo, Zhejiang, China

**Keywords:** effective lens position, intraocular lens, lens power calculation, refractive power, thick lens

## Abstract

**Introduction:**

This study aimed to develop and validate a novel formula for calculating intraocular lens (IOL) refractive power based on Gaussian optics and thick-lens imaging.

**Methods:**

This study was conducted at Ningbo Traditional Chinese Medicine Hospital between October 2021 and October 2023. A total of 54 patients (84 eyes) with age-related cataracts (ARC) undergoing phacoemulsification and IOL implantation were included. The effective lens position was defined as (ACD + W × preoperative LT), where ACD is the anterior chamber depth and LT is the lens thickness. A new IOL power calculation formula was derived using stepwise multiple linear regression, incorporating key ocular parameters including axial length (adjusted for central corneal thickness), keratometry, and the effective lens position. The performance of the new formula was compared with five established formulas: Barrett Universal II, Haigis, Hoffer Q, SRK/T, and Holladay I. For each formula, we compared the mean and median predicted error (PE), the mean and median absolute predicted error (APE), and the proportions of eyes with within ±0.25 D, ±0.50 D, and ±1.00 D.

**Results:**

The newly developed formula demonstrated excellent bias control, with a median prediction error of 0.060 D that was not statistically different from zero (*p* = 0.480). In contrast, the Barrett Universal II (0.450 D, *p* = 0.006), Hoffer Q (0.280 D, *p* = 0.024), and SRK/T (0.515 D, *p* = 0.0004) showed significant hyperopic shifts. The mean error of the new formula (0.071 D) was significantly lower than that of Barrett Universal II, Hoffer Q, and Holladay I formulas (all *p* < 0.01) and comparable to that of the Haigis formula (*p* = 0.226). Its accuracy (mean absolute error, 0.461 D) was comparable to that of all other formulas. The new formula achieved the highest proportion of eyes within ±0.25 D (43.3%), outperforming all other formulas.

**Discussion:**

The proposed IOL calculation formula, which is based on a double-thick-lens imaging model, provides improved control of systematic bias and competitive predictive accuracy. This approach offers a promising framework for clinical applications of personal IOL power calculations.

## 1 Introduction

Intraocular lens (IOL) implantation has undergone substantial advancements in recent decades, transforming cataract surgery into a refractive procedure (1, 2). Achieving accurate postoperative outcomes critically depends on precise IOL power calculations. Despite continuous improvements, predicting the optimal IOL power remains a major clinical challenge. Research has shown that errors in biometric measurements, such as the anterior chamber depth, axial length (AL), and keratometry, can cause errors of 42, 36, and 22%, respectively, in intraocular lens power calculations (3).

Over the past 50 years, IOL power calculation formulas have developed over multiple generations (4). Early formulas were empirically assumed to have a fixed effective lens position (ELP), which resulted in limited accuracy. Subsequent generations incorporated theoretical and regression-based adjustments, progressively improving predictive performance by accounting for axial length, corneal curvature, and ELP (5–7). Modern fourth-generation formulas (Olsen and Barrett Universal II) and a newer formula (Hill-RBF) have further enhanced accuracy through advanced modeling techniques (8). However, these formulas have several limitations. First, the most widely used formulas were developed using specific clinical datasets and may show reduced accuracy when applied to independent populations with different ocular biometric profiles (9). Second, important optical parameters, including central corneal thickness (CCT) and lens thickness, are often neglected despite their influence on the optical system of the eye. Age-related changes in these anterior segment parameters are well-documented as ocular aging biomarkers (10). To address these limitations, this study aimed to develop and validate a novel IOL power calculation formula based on the principles of Gaussian optics and thick-lens imaging. By incorporating population-specific anatomical data and refined optical modeling, we sought to improve prediction accuracy and reduce the systematic refractive error. Development and validation were performed in two prospective consecutive case series.

## 2 Materials and methods

### 2.1 Development of the IOL refractive power calculation formula

#### 2.1.1 Study population

This was a prospective consecutive case series involving 33 patients (52 eyes) with age-related cataracts (ARC) who underwent cataract surgery at Ningbo Traditional Chinese Medicine Hospital between October 2021 and March 2023. This study adhered to the Declaration of Helsinki and was approved by the Hospital Medical Ethics Committee (Approval No. AF/SC-06/04.2/20220129). All participants or their guardians provided written informed consent after full disclosure. The inclusion criteria were: patients aged 50–90 years diagnosed with ARC; uncorrected distance visual acuity (UCDVA) equal to or worse than 0.5 (measured using the standard logarithmic visual acuity chart decimal recording method); phacoemulsification and IOL implantation performed by a single proficient surgeon using a Proming A1-UV IOL (Proming Medical Instrument Co., Ltd., Suzhou, China). The exclusion criteria included:

- Active eye diseases, including pterygium, corneal opacities, dry eye, history of glaucoma, shallow anterior chamber, fundus pathology, uveitis, and other ocular conditions
- History of contact lens wear with inadequate removal time impacting corneal curvature measurement accuracy (hard lenses <4 weeks, soft lenses <2 weeks, orthokeratology lenses <4 weeks)
- History of eye surgery or trauma. Poor fixation, small palpebral fissures, or incompatibility
- Complicated, secondary, posterior subcapsular, or congenital cataracts
- Postoperative complications, such as uveitis, posterior capsular opacification requiring YAG laser capsulotomy, or endophthalmitis
- Corneal endothelial cell density <1,500 cells/mm^2^
- Current participation in other research projects

#### 2.1.2 Sample size estimation

The sample size was calculated using the formula: *n* = ( $\frac{u_{\alpha /2}\sigma }{\delta }$ )^2^. Based on a preliminary survey, the AL standard deviation (*σ*) was set to 1.58, and the allowable error (*δ*) was set to 0.49. Substituting these values into the formula yielded an initial sample size of approximately 40 participants. Considering a 20% follow-up loss/dropout rate, the final optimal sample size was adjusted to 48 participants.

#### 2.1.3 Methods

Preoperative evaluations encompassed visual acuity assessment, automated refraction (CR-800; Topcon Corporation, Tokyo, Japan), non-contact tonometry (NCT; CT-800; Topcon Corporation), slit-lamp examination, subjective refraction (CP-770; NIDEK Co., Ltd., Gamagori, Japan), optical biometry (IOL Master 500; Carl Zeiss Meditec AG, Jena, Germany), optical coherence tomography, anterior segment analysis (model 77,000; OCULUS Optikgeräte GmbH, Wetzlar, Germany), corneal endothelial microscopy (CEM-530; NIDEK Co, Ltd.), A-scan ultrasonography (Compact Touch; Quantel Medical, Cournon-d’Auvergne, France) and slit-lamp examination of the lens and fundus.

The surgical procedure involved cataract phacoemulsification combined with IOL implantation. The appropriate IOL refractive power was selected based on the results of five formulas: Barrett Universal II, Haigis, Hoffer Q, SRK/T, and Holladay I.

Postoperative examinations were performed 1 day and 1 week after surgery, including anterior segment examination, NCT, and automated refraction. One month after surgery, evaluations included slit-lamp examination, NCT, automated refraction, subjective refraction, optical biometry (IOL-Master 500), anterior segment analysis, and A-scan ultrasonography.

#### 2.1.4 Statistical methods

Statistical analyses were performed using SPSS software (v19.0; IBM Corp., Armonk, NY, USA). Measurement data (e.g., age, preoperative NCT, UCDVA, white-to-white corneal diameter [WTW], CCT, ACD, LT, AL, simulated keratometry value, postoperative UCDVA, and ELP) are reported as mean ± standard deviation. Pearson correlation analysis was used to assess the relationships between W (representing the ratio of the IOL position within the capsule to the lens thickness) and WTW, AL, ACD, LT, and age. A multiple linear regression equation was established using W as the dependent variable and WTW, AL, ACD, LT, and age as independent variables, using a stepwise regression method. Statistical significance was set at *p* < 0.05.

#### 2.1.5 ELP formula

The ELP formula was defined as:


$$\mathrm{ELP}=\mathrm{ACD}+W\times {\mathrm{LT}}_{\mathrm{pre}},$$

$$\text{where}\;\;\;\;W=(\mathrm{ELP}–\mathrm{ACD})/{\mathrm{LT}}_{\mathrm{pre}}$$

**Figure 1:**
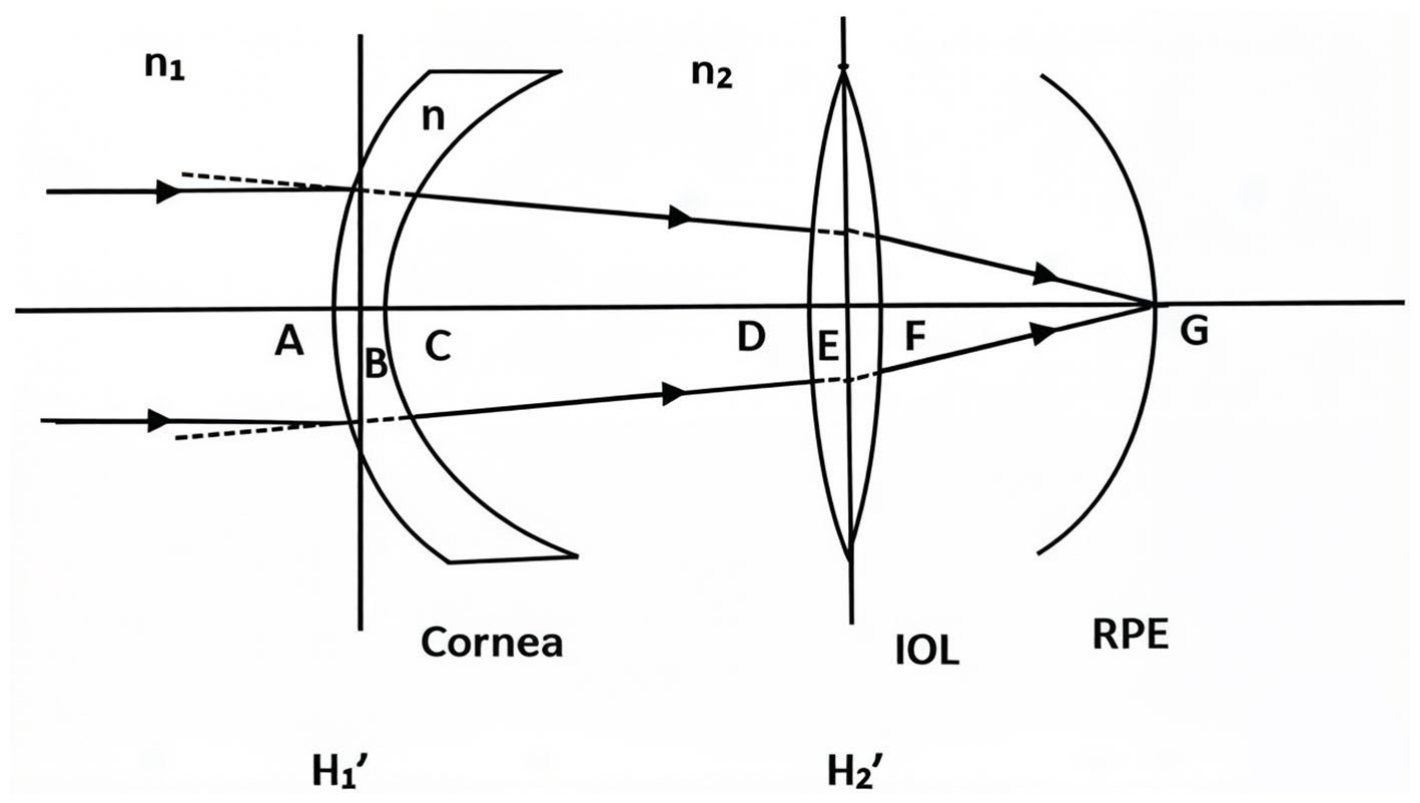
Optical system of the eye with IOL. H1′, H2′ represents the image-side principal plane of the cornea and IOL. The distance BC represents ΔCCT, BE represents ELP_real_, and BG represents AL_real_.

W may be correlated with factors such as WTW, AL, ACD, LT, and age. Regression analysis was used to derive the equation using preoperative and postoperative data. For precision, ELP was adjusted to:


$${\mathrm{ELP}}_{\text{real}}=\mathrm{ELP}–\mathrm{CCT}/2+{\mathrm{LT}}_{\mathrm{IOL}}/2,$$

where *ELP_real_* is the distance between the image-side principal planes of the cornea and IOL, and *LT_IOL_* is the IOL thickness (set at 1 mm due to variations in refractive power) (Figure 1).

#### 2.1.6 IOL refractive power calculation formula


$$P=\frac{1336}{AL_{\text{real}}-\mathrm{EL}P_{\text{real}}}-\frac{1336}{\frac{1336}{\frac{1000}{\frac{1000}{\mathrm{REF}}-12}+K}-\mathrm{EL}P_{\text{real}}}+\mathrm{\Delta P}\;$$

$${\mathrm{AL}}_{\text{real}}={\mathrm{AL}}_{\mathrm{pre}}–\mathrm{CCT}/2$$

$$\mathrm{ELP}=\mathrm{ACD}+W\times {\mathrm{LT}}_{\mathrm{pre}}$$

$${\mathrm{ELP}}_{\text{real}}=\mathrm{ELP}–\mathrm{CCT}/2+0.5,$$

where:

- *P* is the IOL refractive power (D);
- *REF* is the residual refractive power in the spectacle plane (D) with a vertex distance of 12 mm;
- *K* is the average corneal curvature measured using the IOL Master 500;
- *AL_pre_* is the axial length measured using the IOL Master 500 (in mm);
- *AL_real_* is the adjusted axial length (in mm);
- *CCT* is the central corneal thickness measured using A-scan ultrasonography (in mm);
- *ACD* is the anterior chamber depth measured using the IOL Master 500;
- *LT_pre_* is the preoperative natural lens thickness measured using A-scan ultrasonography;
- *W* is the ratio of the IOL position in the capsule to the lens thickness;
- *ELP* is the distance from the anterior surface of the cornea to the anterior surface of the IOL (in mm);
- *ELP_real_* is the distance between the image-side principal planes of the cornea and the IOL (in mm);
- and *ΔP* denotes a correction factor.

### 2.2 Validation of the IOL refractive power calculation formula

#### 2.2.1 Study population

For validation of the newly developed power calculation formula, a prospective consecutive case series was performed including 21 patients (30 eyes) with ARC who underwent surgery at Ningbo Traditional Chinese Medicine Hospital between March 2023 and October 2023. This study adhered to the Declaration of Helsinki and was approved by the Hospital Medical Ethics Committee (Approval No. AF/SC-06/04.2/20220129). All participants or their guardians provided written informed consent after full disclosure. The inclusion and exclusion criteria, pre- and postoperative examinations, and surgical procedure were all identical with those described in Section 2.1.

#### 2.2.2 Statistical methods

The predicted error (PE) was calculated as the difference between the subjective refraction spherical equivalent at 1 month postoperatively and the predicted postoperative residual spherical equivalents calculated using each formula. The mean of the errors is reported as the mean error (ME), and the median of the errors is reported as the median absolute error (MedE). The absolute value of the PE is reported as the absolute predicted error (APE), with the mean of the APE reported as mean absolute error (MAE), the median as median absolute error (MedAE), and the root mean square of the APE as the root mean square absolute error (RMSAE).

Statistical analysis was performed using SPSS software (v 19.0; IBM Corp.). Measurement data such as age, NCT_pre_, UCDVA_pre_ (logMar), WTW_pre_, CCT_pre_, ACD_pre_, LT_pre_, AL_pre_, preoperative simulated keratometry, postoperative 1-month UCDVA (logMar), and postoperative 1-month ELP are expressed as mean ± standard deviation. The ME, MedE, MAE, MedAE, and RMSAE values were calculated for each formula. The PE and APE values of the newly developed IOL calculation formula were compared with those of the other five formulas. Data normality was assessed using the Shapiro–Wilk test. To comprehensively evaluate the performance of different IOL power calculation formulas, an analysis was conducted at two levels:

First, systematic bias was analyzed using the PE (signed) for each formula. All datasets were normally distributed (*p* > 0.05). Repeated-measures one-way analysis of variance was employed to compare the PEs among the six formulas. If the overall difference was significant, Tukey’s multiple comparison test was used for post-hoc pairwise comparisons. Additionally, to determine whether the median PE of each formula differed significantly from the theoretical target value of 0, a one-sample Wilcoxon signed-rank test was performed for the PE of each formula, with the theoretical median set to 0.2. Second, an analysis of accuracy was performed using the APE (the absolute value of PE). Because the APE data of some groups did not follow a normal distribution, the nonparametric Friedman test was applied to compare differences in accuracy among the six formulas. If the overall difference was significant, Dunn’s multiple comparison test was used for post-hoc pairwise comparisons. *p*-values < 0.05 were considered statistically significant for all tests.

## 3 Results

### 3.1 Development of the IOL refractive power calculation formula

Two patients (two eyes) were excluded due to loss to follow-up, leaving 31 patients (50 eyes) included in the study (Table 1). Among them were 12 men (20 eyes) and 19 women (30 eyes).

**Table 1:** Basic data of patients with ARC.

Factor	Mean ± SD	Range
AGE_pre_ (years)	69.40 ± 7.45	55–88
NCT_pre_ (mmHg)	15.88 ± 2.34	12–21
UCDVA_pre_ (logMar)	0.38 ± 0.33	0.05–1.70
WTW_pre_ (mm)	11.21 ± 0.26	10.32–11.67
CCT_pre_ (μm)	552.26 ± 32.98	485–602
ACD_pre_ (mm)	2.90 ± 0.31	2.29–3.65
LT_pre_ (mm)	4.70 ± 0.54	3.51–5.83
AL_pre_ (mm)	23.97 ± 1.79	21.89–30.43
K_pre_ (D)	44.62 ± 1.36	42.17–48.92
Postoperative UCDVA^*^ (logMar)	0.07 ± 0.07	−0.08 to 0.30
Postoperative ELP^*^ (mm)	3.64 ± 0.28	3.03–4.53

^*^All postoperative findings were measured 1 month after cataract surgery.

ARC, age-related cataract; AGEpre, preoperative age; NCTpre, preoperative non-contact tonometry; UCDVApre, preoperative uncorrected distance visual acuity; WTWpre, preoperative white-to-white corneal diameter; CCTpre, preoperative central corneal thickness; ACDpre, preoperative anterior chamber depth; LTpre, preoperative lens thickness; ALpre, preoperative axial length; Kpre, preoperative keratometry; UCDVA, uncorrected distance visual acuity; ELP, effective lens position; SD, standard deviation.

#### 3.1.1 ELP formula

Pearson correlation analysis between W and WTW, AL, ACD, LT_pre_, and age with correlation strengths (from strongest to weakest) of ACD > LT_pre_ > Age > AL > WTW (Table 2). Stepwise multiple linear regression identified ACD and LT_pre_ as independent predictors of W, yielding the regression equation of *W* = 0.973–0.202 × ACD – 0.048 × LT_pre_ (Table 3). The model demonstrated good fit, with an adjusted *R*^2^ of 0.419, and both the coefficients and the model passed significance testing (*p* < 0.05). Therefore, the following ELP formulas were used:


$$\mathrm{ELP}=\mathrm{ACD}+W\times {\mathrm{LT}}_{\mathrm{pre}},$$

$${\mathrm{ELP}}_{\text{real}}=\mathrm{ELP}–\mathrm{CCT}/2+0.5$$

**Table 2:** Pearson regression analysis of W and its possible correlation factors.

Factor	Beta (95% CI)	*t*	*p*
WTW (mm)	0.037 (−0.067, 0.092)	0.329	0.744
AL (mm)	−0.087 (−0.018, 0.009)	−0.656	0.515
ACD (mm)	−0.598 (−0.257, −0.091)	−4.212	<0.001
Age (y)	0.164 (−0.001, 0.005)	1.356	0.182
LT_pre_ (mm)	−0.316 (−0.092, −0.013)	−2.659	0.011

WTW, white-to-white corneal diameter; AL, axial length; ACD, anterior chamber depth; LTpre, preoperative lens thickness; CI, confidence interval.

**Table 3:** Multiple linear regression analysis of W and its possible correlation factors by stepwise regression method.

Factor	Beta (95%CI)	*t*	*P*
Constant	(0.670, 1.276)	6.462	<0.001
ACD (mm)	−0.695 (−0.269, −0.135)	−6.069	<0.001
LT_pre_ (mm)	−0.293 (−0.086, −0.010)	−2.555	0.014

ACD, anterior chamber depth; LTpre, preoperative lens thickness; CI, confidence interval.

#### 3.1.2 IOL refractive power calculation formula


$$P=\frac{1336}{AL_{\text{real}}-\mathrm{EL}P_{\text{real}}}\;\;\frac{1336}{\frac{1336}{\frac{1000}{\frac{1000}{\mathrm{REF}}-12}+K}-\mathrm{EL}P_{\text{real}}}+1.81$$ (1)

$${\mathrm{AL}}_{\text{real}}={\mathrm{AL}}_{\mathrm{pre}}–\mathrm{CCT}/2$$ (2)

$$W=0.973–0.20\mathrm{2ACD}–0.04{\mathrm{8LT}}_{\mathrm{pre}}$$ (3)

$$\mathrm{ELP}=\mathrm{ACD}+W\times {\mathrm{LT}}_{\mathrm{pre}}$$ (4)

$${\mathrm{ELP}}_{\text{real}}=\mathrm{ELP}–\mathrm{CCT}/2+0.5$$ (5)

The correction parameter of 1.81 in the P formula is derived from the mean value obtained by substituting the actual implanted IOL refractive power and the actual postoperative 1-month residual refractive power in the spectacle plane into the P formula Equations (1)–(5).

### 3.2 Validation of the IOL refractive power calculation formula

Two cases (two eyes) were excluded because of failure to follow up, leaving 19 cases (30 eyes) included in the study (Table 4). Among these, there were seven men (12 eyes) and 12 women (18 eyes) subjects. Basic patient data are shown in Table 4.

**Table 4:** Basic data of patients with ARC.

Factor	Mean ± SD	Range
AGE_pre_ (y)	72.27 ± 5.69	61–83
NCT_pre_ (mmHg)	15.07 ± 2.08	12–20
UCDVA_pre_ (logMar)	0.55 ± 0.41	0.10–1.70
WTW_pre_ (mm)	11.26 ± 0.22	10.92–11.80
CCT_pre_ (μm)	557.77 ± 27.90	507–614
ACD_pre_ (mm)	2.94 ± 0.45	2.30–3.80
LT_pre_ (mm)	4.55 ± 0.44	3.68–5.74
AL_pre_ (mm)	23.05 ± 1.00	21.99–27.53
K_pre_ (D)	44.52 ± 1.50	41.87–47.98
Postoperative UCDVA^*^ (logMar)	0.13 ± 0.09	0–0.30
Postoperative ELP^*^ (mm)	3.75 ± 0.33	3.22–4.79

^*^All postoperative findings were measured 1 month after cataract surgery.

ARC, age-related cataract; AGEpre, preoperative age; NCTpre, preoperative non-contact tonometry; UCDVApre, preoperative uncorrected distance visual acuity; WTWpre, preoperative white-to-white corneal diameter; CCTpre, preoperative central corneal thickness; ACDpre, preoperative anterior chamber depth; LTpre, preoperative lens thickness; ALpre, preoperative axial length; Kpre, preoperative keratometry; UCDVA, uncorrected distance visual acuity; ELP, effective lens position; SD, standard deviation.

#### 3.2.1 Accuracy of the formula

Analysis was conducted on all 30 eyes included in the study, with ALs ranging from 21.99 mm to 27.53 mm and an average AL of (23.05 ± 1.00) mm. The accuracies of the IOL refractive power calculations for each formula are listed in Table 5.

**Table 5:** Comparison of accuracy of different formulas.

Factor	New IOL calculation	Barrett universal II	Haigis	Hoffer Q	SRK/T	Holladay I
ME (D)	0.071	0.326	0.165	0.272	0.372	0.338
MedE (D)	0.060	0.450	0.150	0.280	0.515	0.390
MAE (D)	0.461	0.545	0.432	0.549	0.531	0.539
MedAE (D)	0.405	0.525	0.385	0.510	0.540	0.465
RMSAE (D)	0.591	0.622	0.543	0.653	0.618	0.629
±0.25 D	43.40% (13)	20.00% (6)	40.00% (12)	30.0% (9)	26.7% (8)	23.3% (7)
±0.5 D	60.00% (18)	50% (15)	60.00% (18)	50.0% (15)	43.3% (13)	53.3% (16)
±1.00 D	96.67% (29)	86.7% (26)	93.33% (28)	90.0% (27)	90.0% (27)	90.0% (27)

IOL, intraocular lens; ME, mean error; MedE, median error; MAE, mean absolute error; MedAE, median absolute error; RMSAE, root mean square absolute error.

#### 3.2.2 Comparison and analysis of systematic bias (PE) among formulas

##### 3.2.2.1 Inter-formula differences

The PEs of all formulas followed a normal distribution (*P*_1_ = 0.1513, *P*_2_ = 0.1208, *P*_3_ = 0.6155, *P*_4_ = 0.3246, *P*_5_ = 0.3330, *P*_6_ = 0.4050). Repeated-measures one-way analysis of variance indicated a statistically significant difference in PE among the six formulas (*F*[5, 145] = 15.36, *p* < 0.0001). The post-hoc Tukey’s test revealed that the systematic bias of the new IOL formula (ME = 0.071 D) was significantly lower than that of the Barrett Universal II (ME = 0.326 D, *p* < 0.01), Hoffer Q (ME = 0.272 D, *p* < 0.01), and Holladay I (ME = 0.338 D, *p* < 0.001) formulas. No statistically significant difference was found when compared with that of the Haigis formula (ME = 0.165 D, *p* = 0.226).

##### 3.2.2.2 Test for significance of bias

The one-sample Wilcoxon signed-rank test (with 0 as the theoretical median) further elucidated the bias characteristics of each formula. The MedE of the newly developed formula (0.060 D, *p* = 0.480) and the MedE of the Haigis formula (0.150 D, *p* = 0.073) were not significantly different from 0. In contrast, the MedE values of the Barrett Universal II (MedE = 0.450 D, *p* = 0.006), Hoffer Q (MedE = 0.280 D, *p* = 0.024), and SRK/T formulas (MedE = 0.515 D, *p* = 0.0004) were all significantly greater than zero, indicating a tendency towards a hyperopic PE for these formulas.

##### 3.2.2.3 Comparison of accuracy (APE) among formulas

**Figure 2:**
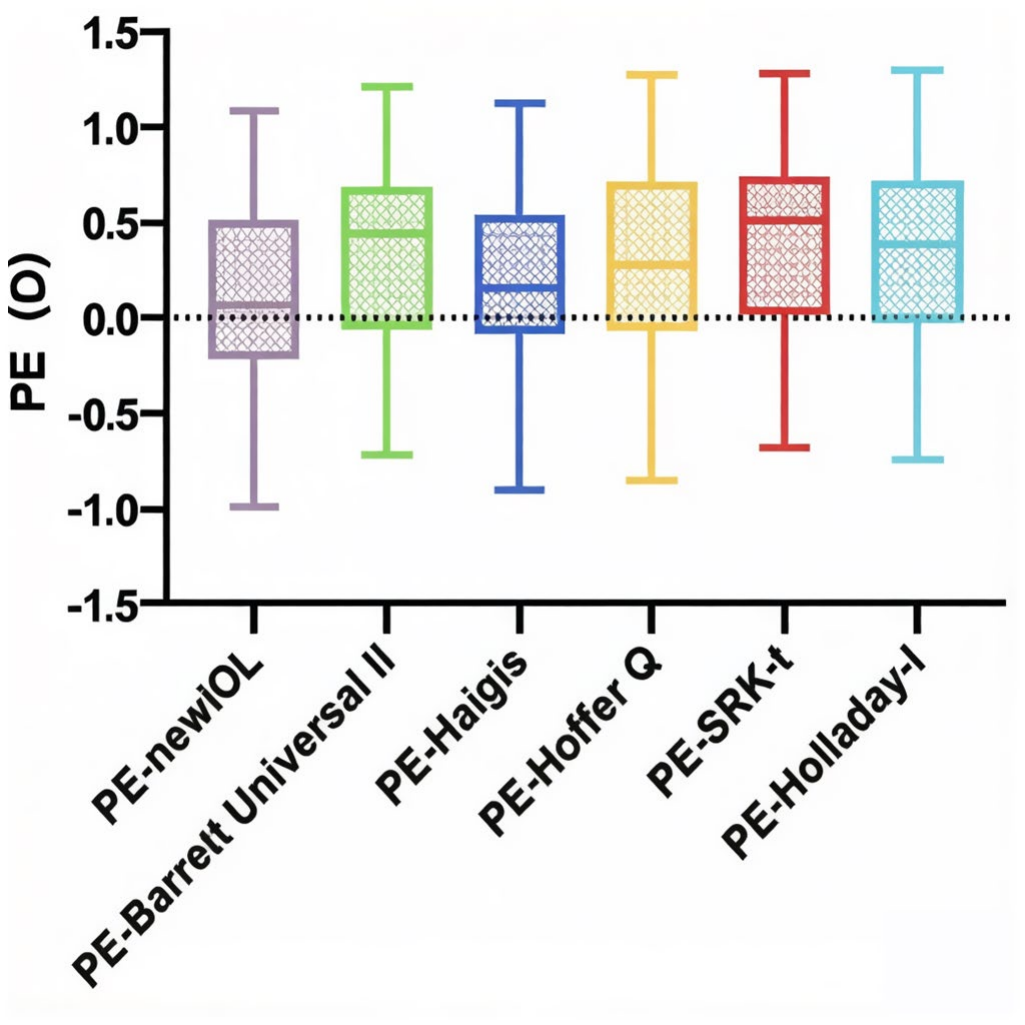
Box plot of the distribution of PE across all groups.

The Friedman test indicated a statistically significant difference in MAE among the six formulas (*χ*^2^ = 15.25, *p* = 0.0094). However, the post-hoc Dunn’s multiple comparison test revealed no significant pairwise differences in MAE between the new IOL formula and any of the other five formulas (all *p* > 0.05). A statistically significant difference was found only between the Haigis (MAE = 0.432 D) and Hoffer Q formulas (MAE = 0.549 D; *p* = 0.0402). Furthermore, the RMSAE among the groups showed no statistically significant difference (*p* = 0.5893, Table 5, Figure 2).

## 4 Discussion

The development of cataract surgery into a refractive procedure has increased the demand for precise IOL power calculation (11). Although advancements in biometry have enabled high precision, the refinement of IOL power formulas remains essential. Over the past 50 years, IOL refractive power calculation formulas have evolved in four generations. In 1967, Fyodorov et al. (12) proposed the first generation of theoretical formulas based on geometric optics in which the ELP was assumed to be 4 mm behind the cornea. However, this results in poor clinical outcomes and significant refractive error. First-generation empirical formulas were derived from a large-scale retrospective analysis of the relationships among postoperative corneal refractive power, AL, refractive status, and emmetropic IOL power. The SRK I formula developed by Sanders et al. using statistical regression methods is the most widely used (13). Although the SRK I demonstrated improved clinical performance over earlier formulas (13), it assumed a constant ACD and fixed the ACD using an A-constant, thereby neglecting posterior IOL movement and limiting predictive accuracy. Second-generation formulas introduced AL dependence with SRK II (14–17), although ELP was still treated as a fixed value. Beginning with third-generation formulas, ELP prediction was improved by incorporating AL, corneal curvature, and other variables. Some formulas have also enhanced prediction accuracy in eyes with extreme ALs, such as the SRK/T, Holladay I, and Hoffer Q formulas (5, 6, 18, 19). Fourth-generation formulas (Holladay II, Haigis, Olsen, and Barrett Universal II) and the newer-generation Hill-RBF formula further emphasize accurate ELP prediction and utilize more sophisticated modeling approaches (8, 20–24).

Despite these advances, some limitations remain, particularly when formulas are applied to populations that differ from the original development cohorts. Most formulas were developed using specific datasets and may show reduced accuracy when applied to independent populations with different ocular biometric profiles (25–33). In addition, optical parameters such as CCT and IOL thickness, which influence the principal planes in the optical system of the eye, are often neglected. These limitations have motivated the development of a formula that integrates population-specific data with enhanced optical modeling.

Our formula introduces two key innovations. First, ELP was defined as ACD + W × LT_pre_, where *W* is the relative position of the IOL within the capsular bag. This formula establishes a mechanistic relationship between postoperative IOL position and preoperative lens anatomy. Regression analysis demonstrated that ACD and LT_pre_ were independent predictors of W (adjusted *R*^2^ = 0.419, *p* < 0.05), yielding the equation *W* = 0.973–0.202 × ACD – 0.048 × LT_pre_. This sample size of 50 eyes satisfied the commonly recommended criteria of at least 5–10 observations per independent variables (34). Second, the model incorporated Gaussian optics by treating the eye as a two-lens system. To enhance physical accuracy, we calculated the distance between the image-side principal plane of the cornea and the object-side principal plane of the IOL (ELP_real_). This was achieved by adjusting the ELP to account for the CCT and assuming a standard IOL thickness of 1 mm, yielding ELPreal = ELP – CCT/2 + 0.5. Using this framework, IOL power was calculated based on the adjusted AL and ELP, and a correction factor (Δ*P* = 1.81) was derived by comparing predicted values with actual postoperative refractive outcomes.

Findings from validation demonstrated that the proposed formula provides improved control of systematic bias. The MedE of 0.060 D was not statistically different from the ideal value of zero, indicating a minimal overall refractive shift, a finding shared only with the Haigis formula (MedE = 0.150 D). In contrast, the other formulas exhibited significant hyperopic shifts. The MAEs of the six formulas were identical, indicating that their accuracies were relatively consistent. Recently, the RMSAE has been used to assess the accuracy of IOL power calculation formulas (35, 36). In our study, there was no significant difference in RMSAE among the six formulas, which implies that the stability and dispersion of the calculation errors were consistent across all formulas. Regarding accuracy, the newly developed formula achieved the highest proportion of eyes within ±0.25 D (43.3%), which is considered a high-precision benchmark. At the commonly used clinical standard of ±0.50 D, it performed comparably to the best-performing established formula (Haigis), with both achieving 60.0% of eyes within this range. Although the absolute accuracy (MAE) was similar across formulas, analysis of the signed PE highlighted the superior ability of the novel formula to minimize systematic bias and achieve the target refraction.

Our findings regarding the performance of the established formulas (Haigis, Hoffer Q, SRK/T, Holladay I) align with previous reports by Hoffer et al. (18) and Du et al. (37). Accurate IOL power calculations are critical not only for cataract surgery but also for phakic IOL implantation in highly myopic eyes, as demonstrated by Wu et al. (38). The performance of the Barrett Universal II formula, a benchmark fourth-generation formula, warrants specific discussion. While showing robust overall accuracy (no significant difference in MAE compared to our formula), it demonstrated a significant systematic bias (MedE = 0.450 D), indicating a hyperopic PE shift. The incorporation of CCT and ELP appears to have contributed to improved bias control. Another advantage of the novel formula is its transparency. Unlike proprietary formulas such as the Barrett Universal II, the present model is fully described, including assumptions, regression-derived parameters (e.g., the equation for W), and the final calculation framework. This transparency facilitates reproducibility and provides a foundation for further refinement and adaptation (39, 40).

This study had several limitations. Although statistically sufficient for model development, the single-center design and moderate sample size may limit generalizability. The IOL thickness was assumed to be constant owing to limited manufacturer data, which may have introduced minor inaccuracies. Additionally, validation was performed using a single IOL model. Furthermore, the inclusion of data from both eyes in some patients, which was not in line with Hoffer’s recommendations, may have introduced a potential statistical bias. Further, the prediction accuracies in the present study differed from those reported in the literature. Some studies indicate that, for currently mainstream IOL calculation formulas, the overall proportion of eyes with a PE within ±0.5 diopters (D) can reach 73.7% (41). However, in the validation dataset herein, even the best-performing formula had a proportion of eyes with PE within ±0.5 D of only 60.0%, which is significantly lower. The sample size in the validation study herein was relatively small, which may have increased the randomness of the results and reduced statistical stability. Further, although the sample in this study covered a wide range of ALs, from short (21.99 mm) to long (27.53 mm), it was not validated by grouping according to AL. Future research should expand the sample size and conduct stratified analyses for different AL subgroups (e.g., short, medium, and long) to evaluate the performance of the new formula more comprehensively and accurately. Finally, this study used only one IOL model (Proming A1-UV), which may limit the generalizability of the formula. Future studies should include large-scale multicenter validations across diverse populations. Incorporating precise IOL thickness measurements and optimizing the constants for different IOL models may further improve accuracy.

## 5 Conclusion

We developed and validated a novel IOL power calculation formula based on Gaussian optics and population-specific ELP estimations. Our new IOL power calculation formula performed better in controlling systematic bias, whereas its absolute error accuracy was comparable to those of existing formulas. This study provides a practical framework for developing transparent and adaptable IOL calculation models that support personalized refractive outcomes in cataract surgery.

## Data Availability

The raw data supporting the conclusions of this article will be made available by the authors, without undue reservation.
